# Hypergammaglobulinemia in the pediatric population as a marker for underlying autoimmune disease: a retrospective cohort study

**DOI:** 10.1186/1546-0096-11-42

**Published:** 2013-11-01

**Authors:** Mindy S Lo, David Zurakowski, Mary Beth F Son, Robert P Sundel

**Affiliations:** 1 Division of Immunology, Department of Medicine, Boston Children’s Hospital and Department of Pediatrics, Harvard Medical School, 300 Longwood Avenue, Fegan 6, Boston, MA 02115, USA; 2 Departments of Anesthesia and Surgery, Boston Children’s Hospital and Harvard Medical School, 300 Longwood Avenue, Boston, MA 02115, USA

## Abstract

**Background:**

The significance of hypergammaglobulinemia as a marker of immune activation is unknown, as a differential diagnosis for hypergammaglobulinemia in children has not been adequately established. The goal of this study was to identify conditions associated with hypergammaglobulinemia in children, with the hypothesis that elevated immunoglobulin levels may precede or predict the development of autoimmune conditions.

**Methods:**

We reviewed the medical records for all children with IgG level ≥2000 mg/dL treated at a tertiary care children’s hospital from January 1, 2000 through December 31, 2009. We compared clinical and laboratory features of these patients, and developed an algorithm to predict the likelihood of underlying autoimmunity based on these characteristics.

**Results:**

After excluding children who had received IVIG, a total of 442 patients with hypergammaglobulinemia were identified. Of these, nearly half had autoimmune conditions, most frequently systemic lupus erythematosus and lupus-related disorders. Autoimmune gastrointestinal disorders such as inflammatory bowel disease were also common. Infectious diseases were the next largest category of diseases, followed with much less frequency by malignant, drug-related, and other conditions. In comparison with non-autoimmune conditions, patients with autoimmune disease had higher IgG levels, lower white blood cell counts, lower hemoglobin values, and lower C-reactive protein (CRP) levels. Multivariable logistic regression confirmed that CRP (P = 0.002), white blood cell count (P < 0.001), hemoglobin (P = 0.015), and female gender (P < 0.001) are independent risk factors for autoimmune disease in patients with high IgG levels.

**Conclusions:**

In a cohort of pediatric patients at a tertiary care children’s hospital, hypergammaglobulinemia was most commonly associated with autoimmune diseases. In female patients with hypergammaglobulinemia, the presence of leukopenia, anemia, and normal CRP was 95% predictive of underlying autoimmune disease.

## Background

Polyclonal hypergammaglobulinemia in adult patients is most frequently related to liver disease, malignancy, autoimmune disease, or infection [1]. The differential diagnosis and clinical significance of hypergammaglobulinemia have not been similarly well defined in children. Malignancies and autoimmunity are much less common in children compared to the adult population, and therefore the implications of an elevated immunoglobulin level in pediatric patients may be different than in adults. Nonetheless, in one study of children with prolonged fever of unknown origin, elevated gamma globulin levels were associated with an ultimate diagnosis of autoimmune disease [2]. In contrast, other more common tests such as blood counts and urinalysis were of comparatively little value in determining the cause of a protracted, unexplained fever.

In children with cryptogenic inflammation, the presence of hypergammaglobulinemia might have clinical implications that hint at diagnosis and/or prognosis. Autoimmune conditions in particular may be difficult to diagnose in children due to their infrequency in the pediatric population and the variability of clinical presentation. We hypothesized that hypergammaglobulinemia might aid in the diagnosis of these conditions. In this study, we examined a cohort of patients treated at a tertiary care children’s hospital over a 10 year period who were found to have significantly elevated IgG levels. We sought to determine diagnoses associated with hypergammaglobulinemia, and to identify risk factors for the presence of autoimmune disease.

## Methods

### Study group

We queried the central laboratory database at Boston Children’s Hospital, a large tertiary care pediatric hospital, for all elevated IgG results between January 1, 2000 and December 31, 2009. All inpatient and outpatient serum IgG levels ≥2000 mg/dL were identified. This cut-off was selected as it represents roughly two standard deviations above the mean at our institution. A single investigator (ML) abstracted relevant information for each patient from the electronic medical record. For patients who had more than one test result above the 2000 mg/dL threshold, data from the earliest encounter were used. The study was approved by the Boston Children’s Hospital Institutional Review Board.

### Consent

As a retrospective study covering a 10 year period of time, contacting patients to consent to medical record review was felt to be impractical. Following data extraction, all information was de-identified to protect patient privacy. Waiver of consent was granted by our institutional review board.

### Data extraction

For all subjects we recorded previously known diagnoses at the time of the test showing an elevated IgG level, new diagnoses made within 1 month of testing, and last recorded diagnosis. Follow up was limited to the most recent document available in the electronic medical record.

Demographic information including age, sex, race/ethnicity, and family history was also recorded. Race and ethnicity were based on self-identified categories from the patient registration database. Presenting complaints, co-morbid conditions, and, when available, additional laboratory data from the day on which the index IgG result was obtained, were also recorded. These values included serum IgA, IgM and complement levels, erythrocyte sedimentation rate (ESR), C-reactive protein (CRP), and complete blood counts.

### Statistical analysis

Univariate analysis included Student’s *t*-test to compare autoimmune and non-autoimmune cohorts for normally distributed laboratory variables (including IgG, ESR, white blood cell count (WBC), absolute neutrophil count (ANC), absolute lymphocyte count (ALC), hemoglobin, and platelet count). The Mann–Whitney *U*-test to compare CRP values since they did not conform to a normal distribution. Multivariable logistic regression analysis (stepwise backward selection) was used to identify significant independent predictors of autoimmune disease based on variables significant in the univariate analysis along with gender as a covariate tested in the multivariate model. Receiver operating characteristic (ROC) curve analysis was applied to determine optimal cut-off values for significant multivariable predictors [3]. Multivariable logistic regression was performed again using the variables with the optimal cut-off values to obtain adjusted odds ratios (OR), 95% confidence intervals (CI) and likelihood ratio tests (LRT) and to develop a predictive algorithm based on the combination of risk factors [4].

Statistical analysis was conducted using IBM SPSS Statistics (version 21.0, IBM, Armonk, NY). Two-tailed values of P < 0.05 were considered statistically significant. Power analysis indicated that a minimum of 150 patients with autoimmune disease and 150 with non-autoimmune disease would provide 80% power to detect an odds ratio of 2.0 using logistic regression analysis for any laboratory test with the established cut-off value (version 7.0, nQuery Advisor, Statistical Solutions, Saugus, MA) [5].

## Results

### Study population

A total of 26,354 patients had a documented IgG level during the study period. There were 1519 results with IgG ≥ 2000 mg/dL belonging to 748 individual patients. For 8 patients, there was insufficient information in the electronic medical record to meet inclusion criteria for the study. Another 298 patients were excluded because they had received IVIG within 1 month of the test result. Among these, common reasons for receiving IVIG included Kawasaki disease, immunodeficiency, idiopathic thrombocytopenic purpura, autoimmune hemolytic anemia, and juvenile dermatomyositis.

The remaining 442 patients were categorized according to their last known primary diagnosis (Table 1). The average age in our study group was 14.3 years (median 14.3, IQR 10.4-17.5); the majority (63%) were female. Racial background was recorded as follows: 229 (58%) identified themselves as “white,” 81 (18%) as “black,” 31 (7%) as Hispanic, and 26 (6%) as Asian. The remaining 75 patients (17%) were identified as “other,” or no racial information was available. The average follow-up period for subjects was 3.48 years (median 2.92).

**Table 1:** Clinical characteristics of patients with hypergammaglobulinemia

	**Total**	**Autoimmune**	**Infection**	**Malignancy**	**PID**	**Drug**	**Other**
	**n = 442**	**n = 221**	**n = 163**	**n = 10**	**n = 15**	**n = 9**	**n = 25**
Median age, years	**14.3**	13.8	14.8	13.2	13.7	16.6	13.8
Female, n (%)	**228 (63)**	168 (76)	80 (49)	2 (20)	11 (73)	5 (56)	12 (48)
Race/ethnicity, n (%)							
White	**229 (52)**	95 (43)	107 (66)	5 (50)	6 (40)	8 (89)	8 (32)
Black	**81 (18)**	49 (22)	24 (15)	2 (20)	0 (0)	0 (0)	7 (28)
Hispanic	**31 (7)**	19 (9)	9 (6)	1 (10)	0 (0)	0 (0)	2 (8)
Asian	**26 (6)**	13 (6)	6 (4)	1 (10)	3 (20)	0 (0)	3 (12)
Other/unknown	**75 (17)**	44 (20)	17 (10.6)	2 (20)	6 (40)	1 (17)	5 (20)
Median follow-up period, years	**2.9**	3.3	3.0	3.5	2.6	0.5	2.0
Death during follow-up period, n (%)	**42 (10)**	6 (3)	30 (18)	3 (30)	1 (7)	0 (0)	1 (4)

*PID* primary immunodeficiency.

### Associated diagnoses

Primary diagnoses were divided into six categories that have been associated with hypergammaglobulinemia in previous reports: 1) autoimmune and autoinflammatory diseases, 2) infections, 3) drug-associated syndromes, 4) malignancies/lymphoproliferative disorders and hematopoietic stem cell transplants, 5) primary immunodeficiencies, and 6) other conditions not fitting these categories. Over 60 unique diagnoses were recorded in association with high IgG levels (Table 2).

**Table 2:** Conditions associated with high IgG level in a pediatic cohort

**Autoimmune/autoinflammatory (221)**	**Infectious (163)**	**Malignancy/stem cell transplantation (10)**
*Lupus/MCTD/SS (82)*	*Chronic/recurrent (88)*	Acute lymphoblastic leukemia
Dicoid lupus	Atopic dermatitis with superinfection	Burkitt lymphoma
Mixed connective tissue disorder	Chronic sinusitis	Castleman’s disease
Systemic lupus erythematosus	Chronic lung disease	Chronic myelogenous leukemia
Sjogren syndrome	Cystic fibrosis	Hodgkin lymphoma
*Arthritis (60)*	*Suppurative (11)*	JMML
Polyarticular JIA	Abscess	GVHD after HSCT
Psoriatic arthritis	Pyogenic pneumonia	Neuroblastoma
Systemic onset JIA	*Streptococcus-associated (22)*	
Spondyloarthritis	Acute rheumatic fever	**Immunodeficiency (15)**
*Vasculitis (12)*	Post-strep arthritis	Autoimmune lymphoprolif. syndrome
Polyarteritis nodosa	Post-strep glomerulonephritis	Autoimmune polyglandular syndrome
Takayasu’s arteritis	Streptococcal pneumonia	Ataxia telangiectasia
Granulomatosis with polyangiitis (GPA)	Streptococcal sepsis	Congenial neutropenia
Eosinophilic GPA	*Viral Immune Activation (19)*	Hyper IgE syndrome
*Gastrointestinal (39)*	CMV	IgA deficiency
Autoimmune hepatitis	EBV	NK cell dysfunction
Crohn’s disease	HIV	
Ulcerative colitis	*Other (23)*	**Drug-associated (9)**
Primary sclerosing cholangitis	Brucellosis	Acute drug hypersensitivity reaction
*Other Autoimmune (28)*	Endocarditis	Drug-induced autoimmunity
Acute demyelinating encephalomyelitis	Lyme disease	
Autoimmune hemolytic anemia	Osteomyelitis	**Other (25)**
Anti-phospholipid syndrome	Tuberculosis	Cirrhosis
Idiopathic thrombocytopenic purpura	Urinary tract infection	Hemoglobin SS
Juvenile dermatomyositis	Viral encephalitis	IgA nephropathy
Kawasaki disease		Liver steatosis
NOMID		Lymphatic malformation
Sweet syndrome		Orthostatic proteinuria
Undefined autoimmune condition		Portal hypertension
		Spastic cerebral palsy trisomy 21

Number of diagnoses are indicated in parentheses.

*Abbreviations*: JIA=juvenile idiopathic arthritis, CMV=cytomegalovirus, EBV=epstein-barr virus, JMML=juvenile myelomonocytic leukemia, GVHD=graft-vs.-host disease, HSCT=hematopoietic stem cell transplant, NOMID=neonatal onset multisystem inflammatory disease.

### Autoimmune and autoinflammatory diseases

The largest category of associated diagnoses was autoimmunity (221 patients, 50%), and rheumatologic diseases dominated this group. Systemic lupus erythematosus (SLE) was the most common diagnosis (48 patients), followed by mixed connective tissue disease (MCTD, 29 patients), polyarticular juvenile idiopathic arthritis (29), systemic onset juvenile idiopathic arthritis (15), and spondyloarthritis (11). Vasculitis diagnoses included Takayasu arteritis, polyarteritis nodosa, cutaneous vasculitis, granulomatosis with polyangiitis (previously known as Wegener's granulomatosis), and eosinophilic granulomatosis with polyangiitis (previously known as Churg-Strauss syndrome). One patient had Kawasaki disease with elevated IgG level noted prior to IVIG treatment.

At the time that a high IgG level was first documented, 15 subjects had so-called 'incomplete lupus,’ with fewer than the 4 criteria necessary to meet American College of Rheumatology criteria for a classification of SLE [6]. Presenting symptoms variably included Raynaud phenomenon, fever, fatigue, arthralgias, rash, pericarditis, and thrombocytopenia. Median follow-up period for these patients was 3.7 years, during which time 6 of the 15 patients eventually met criteria for a rheumatologic condition (2 each MCTD, SLE, and Sjogren syndrome). Another patient developed hypothyroidism 5 years after hypergammaglobulinemia was first detected. Median IgG level when first tested was higher among patients who progressed to a full-fledged autoimmune disease compared to those who did not, although the difference was not statistically significant (2305 mg/dL vs. 2160 mg/dL respectively; P = 0.33). Four of the 6 patients who progressed to a specific rheumatologic diagnosis showed increasing IgG levels over time. In contrast, the IgG level declined over time in all patients who were not ultimately diagnosed with a rheumatologic disease.

A large proportion of the patients in this category had gastrointestinal disorders (39 patients, 18%). There were 14 patients with ulcerative colitis, 10 with Crohn disease, 10 with autoimmune hepatitis, and 9 with primary sclerosing cholangitis (PSC). Several patients had features of more than one gastrointestinal disorder, with the most common overlap being PSC with ulcerative colitis. Two patients with Crohn disease also had spondyloarthropathy, while one patient with ulcerative colitis also had chronic nonbacterial osteomyelitis.

Additional conditions observed in the autoimmune/autoinflammatory cohort included anti-phospholipid syndrome, autoimmune hemolytic anemia (AIHA), idiopathic thrombocytopenic purpura (ITP), acute disseminated encephalomyelitis, juvenile dermatomyositis, and reactive arthritis. One patient had neonatal onset multisystem inflammatory disease (NOMID), an inherited periodic fever syndrome associated with chronic inflammation. Of note, while autoimmune hematologic disease was one of the presenting features for 2 of the patients with partial lupus described above, several other subjects with AIHA or ITP were ANA negative and did not develop other features of SLE during the study period.

### Infectious diseases

The second largest category among children with hypergammaglobulinemia was infection (163 patients, 37%), which could be subdivided into several distinct groups. Of these, chronic or recurrent bacterial infection was the most common (88 patients), the large majority of whom were patients with cystic fibrosis (CF). Streptococcus-related disorders comprised the next most common group (22 patients), with diagnoses including acute rheumatic fever, post-streptococcal arthritis, post-streptococcal glomerulonephritis, and acute streptococcal pneumonia. Chronic viral infections were also associated with hypergammaglobulinemia (19 patients); examples include human immunodeficiency virus (HIV), Epstein-Barr virus, and human cytomegalovirus. One patient with HIV also had Burkitt lymphoma. Finally, 11 patients were diagnosed with suppurative infections, including abscess or pyogenic pneumonia. Additional infectious diagnoses were noted with much less frequency (Table 2).

### Primary immunodeficiencies

The remaining diseases associated with hypergammaglobulinemia were far less common. There were 15 patients with primary immunodeficiencies; as noted earlier, patients receiving IVIG were excluded from this analysis. Diagnoses included both those associated with chronic/recurrent infection (eg. IgA deficiency), as well as those associated with autoimmunity and inflammation (eg. autoimmune polyglandular syndrome).

### Malignancies/lymphoproliferative diseases and hematopoietic stem cell transplants

Six patients were diagnosed with malignancies or lymphoproliferative diseases. Of these, two patients were initially evaluated in rheumatology clinic for unexplained inflammation before their oncologic diagnoses were confirmed.

An additional 4 patients had undergone hematopoietic stem cell transplantation; indications included acute lymphoblastic leukemia, chronic myelogenous leukemia, hemophagocytic lymphohistiocytosis, and neuroblastoma. In some cases the hypergammaglobulinemia was attributed by providers to graft-versus-host disease, while in others the source was not determined.

### Drug reactions

Acute drug hypersensitivity reactions were associated with hypergammaglobulinemia in 2 patients (one each due to carbamazepine and minocycline). Another 7 patients had chronic drug-related autoimmunity, of which almost all were related to minocycline exposure.

### Non-classifiable diagnoses

Twenty-five patients (6%) did not fit any of the above categories. Five of these patients had primary liver dysfunction unrelated to autoimmunity. For the others, however, there was no clear explanation for their elevated gamma globulin levels. These patients included both healthy children as well as those with medical problems not clearly related to immune activation. This latter group included patients with hemoglobinopathies, trisomy 21, and spastic cerebral palsy without known genetic diagnoses. None of these patients had a history of frequent infections as detailed in the medical record, although chronic or subacute infections could not be ruled out. Reasons for checking IgG levels in these patients varied but included fever, fatigue, and elevated ESR.

### Risk of autoimmunity in patients with hypergammaglobulinemia

As nearly half of the patients in our cohort had underlying autoimmune conditions, we sought to define predictive risk factors for the presence of autoimmunity in patients with hypergammaglobulinemia. Univariate analyses showed that patients with autoimmune conditions had higher IgG levels but also lower white blood cell counts (WBC) and hemoglobin (Hgb), hematocrit, complement and CRP levels (Table 3). ROC analysis with the Youden index was applied to determine the optimal values for identifying patients most likely to have autoimmune causes of their hypergammaglobulinemia. Values of <5 mg/dL (50 mg/L) for CRP, <10 mg/dL (100 mg/L) for Hgb, and <5 × 10^3^/μL (5 × 10^9^/L) for WBC were identified. Based on these demarcations, multivariable stepwise logistic regression modeling confirmed that all three were highly significant independent predictors of autoimmune disease, along with female gender (Table 4). The odds ratios and 95% CIs provide measures of risk for each significant predictor in the multivariable model. To provide a clinically meaningful assessment of risk, these variables were modeled in combination to develop a predictive algorithm for estimating the probability of an autoimmune disease stratified by gender (Table 5). Each combination of laboratory tests using the optimal cut-off values is included to highlight the risk stratification for each gender. Among female patients with hypergammaglobulinemia, the presence of anemia, leukopenia, and normal CRP values together was 95% predictive for the presence of autoimmune disease.

**Table 3:** Univariate comparison of laboratory values for autoimmune and non-autoimmune conditions

**Variable**	**Autoimmune**	**Non-autoimmune**	**P value**
	**(n = 221)**	**(n = 221)**	
IgG, mg/dL	2458 ± 680	2325 ± 476	0.018
ESR, mm/hr	63.9 ± 32.8	65.8 ± 32.8	0.675
CRP, mg/dL	0.6 (0.2-2.4)	2.6 (0.8-5.4)	<0.001
WBC, × 10^3^/uL	7.7 ± 4.1	10.7 ± 5.4	<0.001
ANC	4.9 ± 3.3	7.1 ± 4.6	<0.001
ALC	1.9 ± 1.1	2.6 ± 1.7	<0.001
Hemoglobin, mg/dL	11.0 ± 1.7	11.8 ± 2.0	<0.001
Platelets, × 10^3^/μL	359 ± 195	371 ± 150	0.529

Data are mean ± SD, except CRP, which is median (interquartile range).

**Table 4:** Significant independent multivariable risk factors of autoimmune/autoinflammatory disease

**Risk factor**	**Odds ratio**	**95% CI**	**LRT**	**P value**
HgB < 10 mg/dL	2.1	1.2 – 3.8	5.96	0.015
WBC < 5 ×10^3^/μL	3.9	1.9 – 8.0	16.32	<0.001
CRP < 0.5 mg/dL	2.3	1.4 – 3.8	9.81	0.002
Female gender	3.0	1.8 – 4.8	18.45	<0.001

*Abbreviations*: *CI* confidence interval, *LRT* likelihood ratio test.

**Table 5:** Predictive algorithm for the probability of autoimmune/autoinflammatory disease based on combinations of independent risk factors, stratified by gender

			**Probability of autoimmune/autoinflammatory disease**	
**HGB < 10 mg/dL**	**WBC < 5 ×10**^**3**^**/μL**	**CRP < 0.5 mg/dL**	**Female**	**Male**
**-**	**-**	**-**	48%	24%
**+**	**-**	**-**	66%	40%
**-**	**-**	**+**	68%	42%
**-**	**+**	**-**	79%	56%
**-**	**+**	**+**	82%	60%
**+**	**+**	**-**	88%	72%
**+**	**-**	**+**	90%	74%
**+**	**+**	**+**	95%	86%

Probabilities were determined by multivariable logistic regression analysis.

## Discussion

We present here the largest study of children with elevated serum levels of IgG. To our knowledge, this is the first attempt to compile an inclusive differential diagnosis for hypergammaglobulinemia in the pediatric population. The vast majority (almost 95%) of patients in our study had identifiable disorders that could explain their hypergammaglobulinemia, and these conditions could be subdivided into clinically relevant categories. Of these, autoimmune/autoinflammatory disorders were by far the most common.

Hypergammaglobulinemia is well-recognized as a key feature of SLE and lupus-related conditions [7]. The diffuse B cell activation and autoantibody production that is characteristic of SLE is also central to disease pathogenesis. Both MCTD and Sjogren’s syndrome are thought to have related pathogenic mechanisms. SLE and MCTD were the most common diseases observed in this study, representing 17% of the children with hypergammaglobulinemia. While other rheumatologic conditions, such as forms of juvenile arthritis, were also diagnosed relatively frequently, the number with SLE and MCTD was disproportionate to the overall prevalence of these disorders in the pediatric population (approximately 3–12 per 100,000, less than 1/4 of the prevalence of juvenile arthritis [8,9]). Autoimmune conditions of the gastrointestinal tract were also common, in particular inflammatory bowel disease and autoimmune hepatitis.

The infectious conditions associated with high IgG levels also fell into distinct categories. Chronic respiratory infections, such as those seen in patients with CF, were the most common infection associated with high IgG levels. Hypergammaglobulinemia was first noted in CF patients almost 40 years ago [10], and IgG levels correlate with disease progression [11,12]. Streptococcal-associated conditions were also common in our cohort, particularly non-suppurative complications of streptococcal infection (rheumatic fever, post-streptococcal glomerulonephritis, and post-streptococcal arthritis). Hypergammaglobulinemia has long been noted in patients with acute rheumatic fever, and was described at almost the same time that IgG was first characterized [13]. While IgG levels do not seem to correlate with severity of disease, hypergammaglobulinemia may help distinguish active rheumatic fever from inactive rheumatic heart disease [14]. The reason for the uniquely potent serologic response to streptococcal infections is unclear, though streptococcus has a propensity for causing a wide variety of post-infectious sequelae. Among these, Henoch-Schonlein purpura and polyarteritis nodosum were identified only rarely in the present cohort.

Gammopathy, both monoclonal and polyclonal, has been described in adults with a variety of chronic liver conditions [15-17]. In our pediatric cohort, liver disease was a relatively rare association with hypergammaglobulinemia, even when including patients with autoimmune and infectious hepatitis. High IgG levels in the context of autoimmune and infectious liver conditions may reflect chronic immune activation, but the mechanism by which primary liver dysfunction leads to gammopathy in the absence of systemic inflammation is not known. Other factors related to the aging process, or accumulated exposures over time, may explain the discrepancy in the incidence of liver disease among adults and children with hypergammaglobulinemia.

There were only a few patients with malignant conditions in our cohort; all had lymphoproliferative disorders. Two of these had presented to rheumatologists prior to their oncologic diagnosis. The first, a 14 year old girl, had initially been diagnosed with viral arthritis. Additional clinical findings one year later led to the diagnosis of Hodgkin’s lymphoma. The second patient was a 17 year old with multiple physical examination and laboratory criteria consistent with MCTD. However, he was also found to have extensive lymphadenopathy and hepatomegaly. Further evaluation ultimately revealed Burkitt lymphoma. Due to the small number of proliferative disorders in our cohort, we are unable to speculate whether lymphocyte dysregulation led to both hypergammaglobulinemia and development of the malignancy, nor can we identify risk factors for distinguishing autoimmune from malignant conditions. In general, however, malignancy appears to be a rare cause of hypergammaglobulinemia in children.

Given the prevalence of autoimmune conditions in our pediatric cohort with hypergammaglobulinemia, we developed a predictive algorithm for identifying patients who are at increased risk for autoimmunity. We anticipate that this algorithm may be useful in the evaluation of children with unexplained systemic inflammation, since children with hypergammaglobulinemia are most likely to be incidentally identified on the basis of an elevated ESR. While elevated fibrinogen levels cause a high ESR in children with actual inflammation [18], high immunoglobulin levels can also contribute to elevation of the ESR in those with HIV, SLE, and other conditions identified among our study subjects. In such patients, the presence of a low white blood cell count, low hemoglobin, low CRP and female gender should increase concern for a possible autoimmune disease.

Hypergammaglobulinemia in patients with autoimmune/autoinflammatory disorders is multifactorial in etiology. Polyclonal B cell activation is well known to play a prominent role in SLE and other conditions characterized by autoantibodies [19]. Multiple cytokines influence this B cell activation. For example, B cell activating factor (BAFF) and IL-6 are elevated in SLE and other autoimmune diseases [20]. Interestingly, IL-6 has pleiotropic pro-inflammatory effects that also play a role in the pathogenesis of autoinflammatory conditions [21,22]. It is not clear how B cells influence autoinflammatory disorders such as NOMID and Crohn disease; one possibility may be that B cell activation and antibody production in these conditions are bystander effects of IL-6 activity.

A significant limitation of this study is the potential bias among children in whom a decision was made to measure immunoglobulin levels. Rheumatologists and immunologists are more likely to check immunoglobulin levels than other specialists, and thus patients with rheumatologic and immunologic disorders are probably overrepresented in our cohort. On the other hand, studies of hypergammaglobulinemia in adults, and previous case series in children, do not suggest that large numbers of children who have other illnesses or who are entirely well are likely to have IgG levels above 2000 mg/dL. Nonetheless, as our predictive algorithm was developed from this potentially skewed population, it will require prospective testing before its general applicability can be confirmed.

## Conclusions

Significant hypergammaglobulinemia is relatively uncommon among children, with fewer than 500 patients identified with non-iatrogenic IgG levels over 2000 mg/dl at a large, tertiary care children’s hospital over the course of a decade. A significant number of these children were diagnosed with a relatively limited number of serious but treatable conditions. Though the differential diagnosis of a serum IgG level above 2000 mg/dl in children is similar to that described in adults, there are important differences. In particular, autoimmunity is significantly more highly represented, while liver disease and malignancy are relatively infrequent causes of high IgG levels in children. There should be a high index of suspicion for underlying autoimmune disease in pediatric patients with high IgG levels and no clear infectious or malignant process. This risk of an autoimmune disease may be further quantified on the basis of gender, white blood cell count, hemoglobin level and CRP.

## Competing interests

The authors declare that they have no competing interests.

## Authors’ contributions

ML is the corresponding author and participated in the study design, data collection, analysis, and drafting of the manuscript. DZ performed the statistical analysis. MS participated in the interpretation of data and manuscript preparation. RS oversaw the study design, data analysis, and manuscript preparation. All authors read and approved the final manuscript.
